# GelSight: High-Resolution Robot Tactile Sensors for Estimating Geometry and Force

**DOI:** 10.3390/s17122762

**Published:** 2017-11-29

**Authors:** Wenzhen Yuan, Siyuan Dong, Edward H. Adelson

**Affiliations:** Computer Science and Artificial Intelligence Laboratory (CSAIL), Massachusetts Institute of Technology, Cambridge, MA 02139, USA; yuan_wz@csail.mit.edu (W.Y.); sydong@mit.edu (S.D.)

**Keywords:** GelSight, tactile sensing, robotics

## Abstract

Tactile sensing is an important perception mode for robots, but the existing tactile technologies have multiple limitations. What kind of tactile information robots need, and how to use the information, remain open questions. We believe a soft sensor surface and high-resolution sensing of geometry should be important components of a competent tactile sensor. In this paper, we discuss the development of a vision-based optical tactile sensor, GelSight. Unlike the traditional tactile sensors which measure contact force, GelSight basically measures geometry, with very high spatial resolution. The sensor has a contact surface of soft elastomer, and it directly measures its deformation, both vertical and lateral, which corresponds to the exact object shape and the tension on the contact surface. The contact force, and slip can be inferred from the sensor’s deformation as well. Particularly, we focus on the hardware and software that support GelSight’s application on robot hands. This paper reviews the development of GelSight, with the emphasis in the sensing principle and sensor design. We introduce the design of the sensor’s optical system, the algorithm for shape, force and slip measurement, and the hardware designs and fabrication of different sensor versions. We also show the experimental evaluation on the GelSight’s performance on geometry and force measurement. With the high-resolution measurement of shape and contact force, the sensor has successfully assisted multiple robotic tasks, including material perception or recognition and in-hand localization for robot manipulation.

## 1. Introduction

Tactile sensing is an important mode for both human and robots to perceive the environment. In the past decades, researchers have developed many different tactile sensors for robots [1,2,3,4], and the core part of those tactile sensors is to detect the contact and contact force, or force distribution over the fingertip area. For example, a successfully commercialized sensor is the tactile sensor array from Pressure Profile Systems, which measures the normal pressure distribution over the robot fingertip, with a spatial resolution of 5 mm. The sensor has been applied to multiple commercialized robots, including the PR2 robot, and Barrett hands, and it successfully assisted common robotic tasks, such as contact detection and gripping force control. With the force measurement from the fingertip tactile sensors, a robot is much less likely to break delicate objects. The contact detection and localization also refine the robots’ performance in grasping, and in-hand manipulation.

However, tactile sensing can be much more informative for robots. Considering humans as an example, although we are not able to precisely measure contact force, humans can get abundant information from tactile sensing, such as the objects’ shape, texture, materials, and physical properties including mass, compliance, roughness, friction and thermal conductivity [5,6]. Tactile sensing is an important part for humans’ closed-loop manipulation as well. With touch, we can know whether the cup in the hand is going to slip, and thus adjust the gripping force accordingly; we can know whether a USB connector is going to be correctly plugged into the socket because we get the feedback of the impedance force. Those tasks, however, are still challenges for robots because they are not yet able to fully apply tactile sensing. Robots need more than force feedback, while the existing sensors do not obtain enough tactile information for the robots. Thus, we ask the question: what kind of tactile signal is needed for the robots to better understand the environment?

We try to address the question with the answer that geometry sensing is equally important as the force sensing for robots. To better measure the geometry, a deformable surface, and high spatial resolution sensing are required. With the measurement of high-resolution geometry, the robot will be able to learn more about the objects’ shape and texture. Moreover, the dynamic interaction between the soft sensor and the environment can reveal more physical properties of the object being contacted, such as the slipperiness and compliance.

In this paper, we discuss the development of a tactile sensor called GelSight, which measures high-resolution geometry, and which can be used to infer local force and shear. The sensor uses a deformable elastomer piece as the medium of contact, and an embedded camera to capture the deformation of the elastomer surface. The high resolution 3D geometry of the contact surface can be reconstructed from the camera images. When the sensor surface is painted with small black markers, the motion of the markers provides information about both normal force and shear force. The vision-based design of the sensor also makes the hardware accessible and the installation much simpler, and the software for processing the raw data easier to develop by using the algorithms in computer vision. With the help of GelSight, a robot can easily capture the detailed shape and texture of the object being touched, which makes the touch-based object or material recognition much easier. Research also shows that GelSight can also help a robot to sense multiple physical properties of the objects.

The first GelSight prototype was developed in 2009 by Johnson and Adelson [7]. Its high resolution capabilities were further demonstrated in [8]. Unlike other optically based approaches, GelSight works independently of the optical properties of the surface being touched. The ability to capture material-independent microgeometry is valuable in manufacturing and inspection, and GelSight technology has been commercialized by a company (GelSight Inc., Waltham, MA, USA). However, the requirements for metrology are quite different than those of a robot finger.

For a robot finger, it is necessary that the sensor be compact enough to be mounted on an end effector. At the same time, there is no need for micron-scale resolution. We have developed sensors that are compact, yet have resolution far exceeding that of human skin. In this paper, we will firstly demonstrate the basic working principle and software development of the GelSight, and then we will introduce the design and fabrication processes of different sensor versions. Following that, we will show some experiment results on the robotic fingertip GelSight’s performance on measuring geometry and force. For the force measurement, since the sensor displacement, which is directly measured by GelSight, is in the nonlinear relationship to the contact force, we use deep neural networks to measure the contact force from the learned examples, and show some preliminary results of the force measurement with basic shapes. In the last section, we will briefly introduce some examples of GelSight helping robots with multiple tasks.

## 2. Related Work

Some thorough reviews of the existing tactile sensors in the past decades are given by [1,2,3,4]. The sensors use different sensing principles, such as resistance, capacitance, piezoelectricity, optic component, or magnetics. The major focus of the sensors has been measuring the pressure distribution, or contact force and location, over a certain area. For the tactile sensors applied on robots, most of the sensors are designed for fingertip or gripper end-effector (an example is [9]), which measures the force or contact during grasping; some other sensors designed for body (an example is [10]), which detect the contact over a much larger area and are commonly used for contact or collision detection during the robot motion.

Among the tactile sensors, the optical sensors based on vision stand out because they are of easier wiring and fabrication processes, and can mostly provide a relatively high spatial precision in locating contact area. The vision-based tactile sensors usually use a deformable body, either a piece of rubber or a fluid balloon, as the sensing medium, and apply a camera to capture the deformation of the medium. In most cases, the deformation is indirectly measured from other visual cues, such as the deformation of the pre-printed patterns on the medium. In the 1980s, Schneiter and Sheridan [11] and Begej [12] used an optical fiber to capture the light reflection on silicone surface, and used cameras to capture the change of optic signal from the optical fibers. The deformation of the medium would cause a change in the light reflection. A successful example is Tar and Cserey [13], which has already been commercialized. The sensor used a hollow hemispherical rubber dome as the contact medium, the dome has a reflective inside surface. The sensor uses three receivers in the bottom to measure the reflective light from the deformed dome, thus estimating the three-axis contact force. However, the spatial measurement is not available with the sensor.

Another popular design for a vision-based sensor is to print marker patterns on or in the sensing medium, and track the motion of the markers from the embedded camera. Some examples of the designs include [14,15,16,17]. Ferrier and Brockett [14] developed an analytical model to calculate the value and position of the point-contact force on a round shaped fluid finger. However, in most cases, an analytical model is hard to build and is restrained by many contact requirements. The nonlinearity of the elastomer material, and the complicated contact condition with multiple contact points or contact surface greatly increases the difficulty. Kamiyama et al. [15] developed a large flat sensor with two layers of markers in the cover rubber, and they used a simplified mechanical model to calculate normal and shear force during the point contact condition. In their later work [18], they scaled down the sensor design to a robot finger tip. They used experimental methods to calibrate the contact force. Chorley et al. [16] designed another hemispherical sensor with markers close to the surface, and Cramphorn et al. [19] introduced an improved design by adding the core-shaped fingerprint on the sensor surface. The sensor is 3D printed, which makes it much easier to reproduce. Ward-Cherrier et al. [20] showed the sensor can be adjusted to different 3D shapes and applied on robot grippers. TacTip could sensitively discriminate the contact, and can be used to extract the approximate edge of the contact surface. The force was not measured, but the edge detection successfully helped a robot to localize contact and follow the contours [21]. Ito et al. [17] designed a sensor that had a hemispherical shape filled with translucent water, and the marker patterns were printed on the surface. The sensor responded to contact with both marker motion and the change of filling water’s reflection, and they built an analytical model to estimate the shapes when the contact shape is simple. They also showed in [22] that, by counting the markers that were ‘stuck’ on the surface or ‘lagged behind’, the sensor could estimate partial slip stage. However, their sensors are too large in volume to be properly applied on robots.

The high-resolution measurement is still largely under-exploited. Maheshwari and Saraf [23] offered one possible solution: they proposed an electro-optical tactile sensor that used a surface film made of metal and semiconducting nanoparticles that converted pressure into optical signals. The sensor is able to sense the pressure distribution at a resolution similar to human fingers.

Here are some major challenges for making the desired robotic tactile sensors:

- Measurement of shear force. Only some of the tactile sensors are able to measure shear force as well as normal force, while the shear force is very important in multiple robotic tasks, such as estimating object states in grasping, estimating surface friction of the objects through touch.
- Detecting contact area. Most of the sensors focus on the situation of point contact, which means they are designed to measure the location and magnitude of the contact force. However, in many tactile tasks, the contact is an area, or multiple areas, instead of a single point. Sensors that can detect more tactile information based on the contact area are desired.
- Hardware optimization. If the sensors can be used on the robots, they must be small in size, easy on wiring, and offer real-time feedback. Some sensors have delicate structures and good signals, but the design is either too bulky or too complicated to be mounted on robot hands.
- Fabrication challenge. A major work in the research of the tactile sensing is to develop a method to fabricate the sensors, which is usually nontrivial. Unfortunately, most of the fabrication methods are not well shared—it is hard for another lab to duplicate the complicated fabrication procedures. TacTip offers a good example where the 3D printing methods are open sourced. In other cases, devices have been commercialized and are therefore available via purchase. Two good examples are the Optoforce sensor (OptoForce Kft., Budapest, Hungary) [13] and the BioTac sensor (SynTouch Inc., Montrose, CA, USA) [24]: the researchers founded startups to improve the product design and produce the sensor commercially, so that other robotic labs have direct access to the sensors.

The GelSight sensor can precisely locate the contact area, and even measure the high-resolution shapes. It also can be used to estimate shear force and slip state. Apart from the sensing principles, we also introduce our effort in improving the compact design of the sensor and the fabrication process, so that the sensor can be easily applied on robot grippers. We also try to encourage other labs to reproduce the sensor by simplifying the fabrication process, and sharing the 3D printing files and other fabrication details.

## 3. Principle of GelSight

### 3.1. Overview

**Figure 1:**
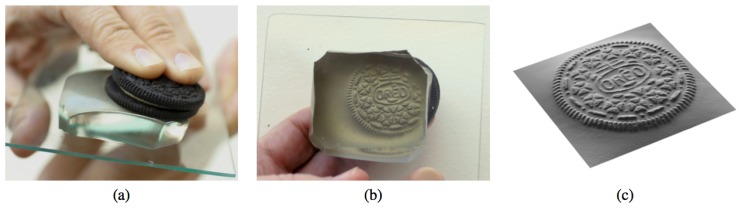
(**a**) a cookie is pressed against the skin of an elastomer block; (**b**) the skin is distorted, as shown in this view from beneath; (**c**) the cookie’s shape can be measured using photometric stereo and rendered at a novel viewpoint [7] (Got copyright permission from IEEE).

The GelSight sensor was initially designed to measure the 3D shape and texture of the target surface, as firstly introduced by Johnson and Adelson [7]. The device consists of a slab of transparent elastomer covered with a reflective coating membrane. When an object is pressed on the elastomer, the membrane distorts to take on the shape of the object’s surface, but with consistent reflectance. When viewed from behind, the membrane appears as a relief replica of the surface. We place a camera to record the image of this relief, using illumination from light sources at different directions. A photometric stereo algorithm [25] is then used to reconstruct the depth map of surface. Figure 1a,b shows an example of an Oreo cookie being pressed against the elastomer, while the reflective membrane takes on the shape of the Oreo’s surface. We reconstruct the surface from the shaded image, which is rendered in Figure 1c. The spatial resolution of the sensor, when optimized for resolution, can reach 1–2 microns. In the case of compact GelSight devices designed for robot fingers, the spatial resolution is typically in the range of 30–100 microns.

**Figure 2:**
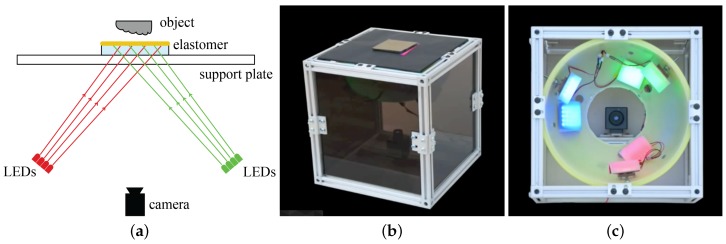
(**a**) basic principle of the Gelsight and the desktop design introduced in [7]. There are four main components for the GelSight sensor: an sensing elastomer piece with the opaque reflective membrane on top, supporting plate, LEDs which provide illumination, and the camera in the bottom to capture the shaded images under the illumination from different directions; (**b**) shows the picture of the sensor, and (**c**) shows the arrangement of the LEDs and camera when viewing from the top.

Photometric stereo is a technique in computer vision for estimating the surface normals of objects by observing the object under different lighting conditions. Theoretically, when the surface reflectance and light properties are known, the reflection under lights from three separate directions can be calculated. For GelSight, the reflectance of the coating membrane is known and consistent, and the lighting condition is well controlled: different LED arrays are arranged at different directions. Images corresponding to the multiple directions can be recorded sequentially, or separated by using different color channels. By combining the shading from three or more directions, we estimate the surface normals on each pixel of the shaded image, and then integrate the surface normal to get the 3D shape of the surface. Figure 2 shows the schematic and pictures of the GelSight device introduced in [7].

**Figure 3:**
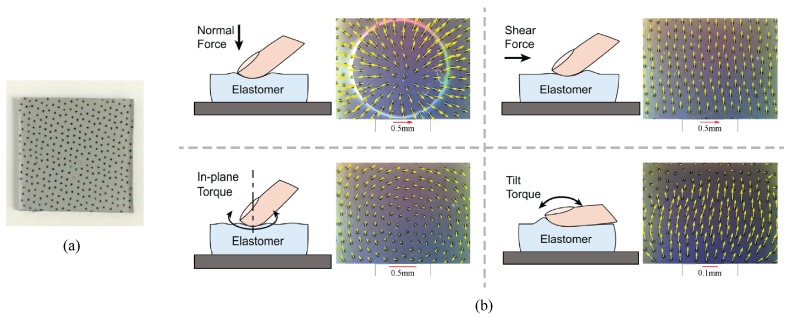
(**a**) an example pattern of printed markers on the GelSight. In the figure, the elastomer is for the fingertip GelSight sensor [30] (Got copyright permission from IEEE), with the dimension of 25 mm × 25 mm × 2 mm, the markers have an average interval of 1.1 mm; (**b**) the markers make diverse motion field patterns under different kinds of forces or torques. The magnitude of the markers’ motion is roughly proportional to the force/torque value.

Photometric stereo provides a height map, i.e., it describes the displacement of the membrane surface along the *z*-axis. During contact, the membrane also moves tangentially. We can measure the tangential displacements (in *x* and *y*) by printing markers on the membrane and tracking their displacement over time [26,27]. We paint the black dots, either evenly or arbitrarily distributed, between the elastomer base and the reflective membrane. Their motion in the camera image directly corresponds to the lateral displacement of the elastomer’s surface, and can indicate contact force and torque, as demonstrated in Figure 3b: different kinds of force or torque will make different patterns of motion field, and the magnitude of the marker motion is roughly proportional to the amount of force or torque. The distribution of the marker motion also tells information about slip or incipient slip states.

We have developed multiple versions of GelSight devices, with similar working principles but different dimensions, illumination arrangements, elastomer shapes and coatings. They have different resolution, speeds, and aim at varied working conditions and applications. Those designs of GelSight are introduced in Section 4. Lately, we developed a compact version for robot grippers, called fingertip GelSight sensors [28,29]. The sensors are compact in volume, and can provide near real-time signal feedback. The elastomer sheets on these sensors are much thinner (under 2.5 mm), and are of domed shape to allow a greater range of contact conditions.

### 3.2. Material

In this section, we introduce how to choose the materials for the sensor’s sensing elastomer and the reflective coating.

For choosing the elastomer base for the sensor, there are several factors that need to be considered: optical transparency, stretchability, hardness, robustness, and complexity to fabricate. We require the elastomer to be optically clear, and stretchable enough to yield to the object shapes, but the hardness could be varied according to different applications. We typically use elastomer with Shore A values between 5 and 20. The common materials we use for making the elastomer are thermoplastic elastomers (TPEs) such as styrenic block copolymers, and silicone rubbers. TPEs are cast by melting, while the silicone rubbers are two-part liquids that cross-link into elastomers.

In general, we wish the sensor elastomer base to be soft enough so that it is sensitive to small contact force. However, if the sensor is to be used in heavy-loaded environments, a harder elastomer base is desired to ensure the elastomer will be sensitive in the measuring range. At the same time, the hardness of the elastomer is restricted to the available elastomer material. As an example, the elastomer in the fingertip GelSight sensor (introduced in Section 4.2) is a clear silicone that is close to a neo-Hookean solid, with the coefficient μ of 0.145 MPa, and the hardness is close to Shore 00-45. In practice, the sensor with this hardness works well for moderate contact perception and robotic manipulation tasks. The minimum perceivable force of the sensor, when contacting different types of objects, is mostly less than 0.05 N. We use an ATI Nano-17 force/torque sensor (ATI Industrial Automation, Apex, NC, USA) to calibrate the force measurement of GelSight (as shown in Figure 14a, and GelSight performs more sensitively than the ATI sensor in detecting small contact forces. The detailed results are shown in Table 1.

**Figure 4:**
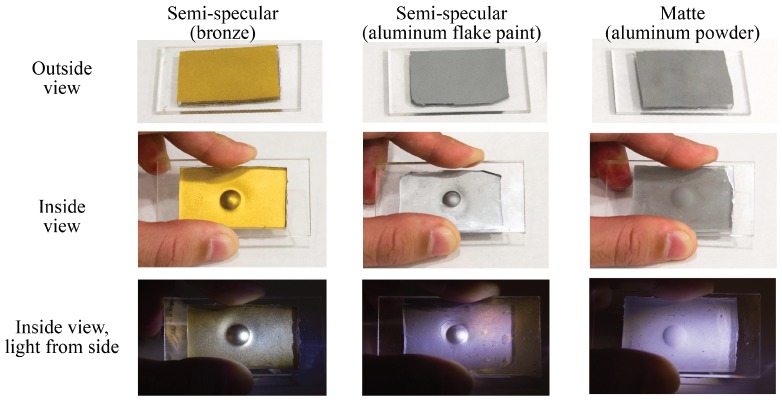
Three kinds of elastomer coatings: semi-specular coating painted by bronze flake and aluminum flake paint, and matte coating by aluminum powder. In the second and third row, the three pieces of elastomer are pressed against a ball with diameter of 6 mm, but the in the 3rd row, the elastomer is illuminated by light from side direction.

The coating skin is a thin layer of silicone mixed with pigment. We choose pigment with different reflective properties for different sensor designs or applications. Examples of the commonly used matte and semi-specular coatings are shown in Figure 4. A semi-specular coating is sensitive to small variations on the surface normal, and a matte coating is favorable for accurate measuring of the general shapes. The different reflective properties requires different optical designs. However, in all cases, the pigment should be made of fine particles, and the coating should be thin in order to reveal the fine details on the object. We use bronze flake or aluminum flake pigment for the semi-specular coating, and fine aluminum powder for the matte coating.

### 3.3. Algorithm for Measuring Shape

We model the surface of the sensor with a height function z=f(x,y), so that the surface normal is $N\left(x,y\right)=(\frac{\partial f}{\partial x},\frac{\partial f}{\partial y},-1)$ (The surface normal is in this form because the surface is the zero level surface of a scalar field ϕ(x,y,z)=f(x,y)−z, and which is normally equal to ∇ϕ). Given the lighting and surface reflection are evenly distributed, the shading depends only on the local surface normal, which means that the cast shadows or internal reflections are neglected. Under this assumption, under the single light source, the light intensity at (x,y) can be calculated as $I\left(x,y\right)=R\left(\frac{\partial f}{\partial x},\frac{\partial f}{\partial y}\right)$. The reflectance function *R* models both the lighting environment and the surface reflectance. Considering the lighting sources from multiple directions, which can be inferred as the different channels in the red-green-blue (RGB) image I when the lights are of different colors, we have

(1) $$I\left(x,y\right)=R\left(\frac{\partial f}{\partial x}\left(x,y\right),\frac{\partial f}{\partial y}\left(x,y\right)\right),$$

where

(2) $$I\left(x,y\right)=(\;\begin{array}{ccc} I_{1}\left(x,y\right) & I_{2}\left(x,y\right) & I_{3}\left(x,y\right) \end{array}\;),R\left(p,q\right)=(\;\begin{array}{ccc} R_{1}\left(x,y\right) & R_{2}\left(x,y\right) & R_{3}\left(x,y\right) \end{array}\;).$$

We use different lights of red, green and blue colors from different directions, so that $I_{1}\left(x,y\right),I_{2}\left(x,y\right)$ and $I_{3}\left(x,y\right)$ correspond to the shading images under the lights from a single direction. We assume R is a consistent function for one sensor.

The reflectance functions R are in the nonlinear relationship to $\left(\frac{\partial f}{\partial x},\frac{\partial f}{\partial y}\right)$, and we are interested in the inverse function—a function that maps observed intensity to geometry gradients. To do this, we build a lookup table. The lookup table is three-dimensional, and each entry contains a gradient and a first-order approximation of the reflectance functions in the neighborhood near the gradient. In a simplified version, we only record the gradient at each entry space and match the gradient reading to the closest entry. The lookup table is generated by a calibration procedure, when a small sphere with known diameter is pressed on the sensor, as introduced in Section 3.5.

After getting $\left(\frac{\partial f}{\partial x},\frac{\partial f}{\partial y}\right)$ using $R^{-1}$, we reconstruct the heightmap z=f(x,y) by integrating the normals. It can be precisely calculated using re-weighted least squares approach (IRLS) [31], as introduced in [8], or can be solved using Poisson equation:

(3) $$\nabla^{2}f=g,$$

where

(4) $$g=\frac{\partial }{\partial x}(\frac{\partial f}{\partial x})+\frac{\partial }{\partial y}(\frac{\partial f}{\partial y}).$$

We solve the Poission Equation (3) using fast Poisson solver with discrete sine transform (DST), thus we can get a fast computation on the heightmap reconstruction, and it is possible to run the algorithm online [32].

### 3.4. Algorithm for Measuring Marker Motion

Apart from getting the heightmap, we also calculate the motion of the markers from the images. In the initial GelSight frame $Frm_{0}$, where nothing is in touch with the sensor, we locate the markers and record their positions $u_{0}$; in the following frames, we also calculate the markers’ positions u, so the motion is $du=u-u_{0}$.

**Figure 5:**
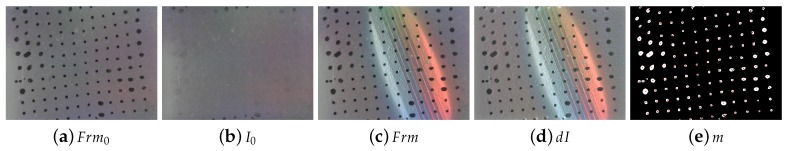
The procedure of segmenting and locating the markers in the GelSight images. (**a**) the initial GelSight frame *Frm*_0_ when noting is in touch; (**b**) the low-pass Gaussian filtered image *I*_0_ from *Frm*_0_, where only the color background is left; (**c**) a GelSight frame *Frm* when the sensor is touching a cylinder; (**d**) the image *dI* after subtracting the initial background *I*_0_ from the current frame *Frm* (color is normalized for display purpose); (**e**) the mask of the markers after thresholding on *dI*. The red dots denotes the centroids of the markers.

Figure 5 shows the procedure of locating markers from Frm: in the beginning, we record the initial frame $Frm_{0}$, and get the image’s background $I_{0}$ using low-pass Gaussian filter on $Frm_{0}$, where the black markers and high-frequency noise are removed. For a given image Frm, we calculate the differential image $dI=Frm-I_{0}$, which shows only the change in the image. In dI, the pixels where the sensor surface has a big curvature and large surface normal will have a large color intensity, but the black markers are dark. We use a threshold on dI to get the black area *m*, which correspond to the area of markers. Then, we calculate the centroids of each connected area in *m*, and consider them as the markers’ positions u.

### 3.5. Calibration for Shape Measurement

The sensor calibration aims to find the mapping between pixel intensities of the GelSight images and the surface normal or geometry. In other words, it builds the lookup table of the function $R^{-1}\left(I\right)$, which is the reverse function of Equation (1). Each GelSight sensor is slightly different in construction, so that, for measuring the shape, the calibration is mandatory.

**Figure 6:**
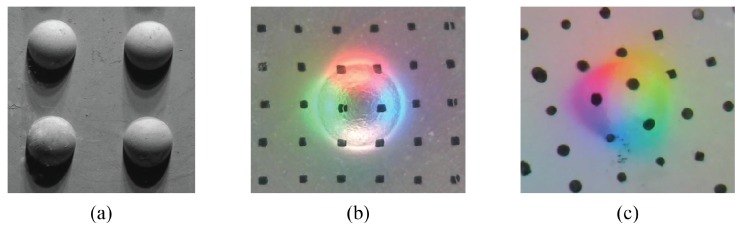
Calibration images (part) with different GelSight sensors. During calibration, a ball/ball array is pressed on the sensor, and the image intensity change corresponds to the surface normal of the ball. (**a**) the calibration image of the side-illuminated GelSight device [8] (Got copyright permission from Association for Computing Machinery), under the single-colored illumination from one direction; (**b**) the calibration image of the first fingertip GelSight sensor [28] (Got copyright from MIT), where the illumination has four colors; (**c**) the calibration image of the improved fingertip GelSight [29] (©2017 IEEE), where the illumination has three colors. For (**b**,**c**), we press the ball at different locations, and take the average color change value to surface normal.

During the calibration, we press a small ball on GelSight, and record the image. Examples of the GelSight images for different sensors are shown in Figure 6. Since the ball is of known shape, we are able to know the surface normal at every pixel. We choose a ball because the hemispherical shape theoretically contains surface normals in all directions. From the image, we can get the correspondence of the pixel intensity I(x,y) to the surface normal $\left(\frac{\partial f}{\partial x},\frac{\partial f}{\partial y},1\right)$. In practice, instead of using the direct intensity *I*, we use the relative intensity $dI=I-I_{0}$ as introduced in Section 3.3, in order to eliminate the influence of the spatial inhomogeneity in the illumination. We make the lookup table $R^{-1}$ as a three-dimensional matrix, corresponding to the three color channels of the input images. For the Fingertip GelSight devices, which use a USB webcam and output the color images in 8 bits, we select the dimension of $R^{-1}$ as 80×80×80.

To further reduce the influence of the markers, noise, and inhomogeneity of the light conditions on the calibration image, we record the multiple images of the ball pressed on various positions on the sensor surface, and make the final look-up table from the average values of all images. For the intensities that are not captured in the images, we use a linear interpolation to fill in the blanks.

### 3.6. Marker Motion for Force Measurement

The markers’ motion represents well the contact force in two ways: the pattern of the motion field indicates the type of the force or torque, and the magnitude of the motion is roughly proportional to the force [26,27]. Examples of the different motion field patterns are shown in Figure 3: under the normal force, the markers spread outwards from the contact center; under the shear force, the markers all move in the shear direction; under the in-plane torque, which means the torque axis is perpendicular to the surface, the markers move in a rotational pattern. When there is a combination of multiple force types, the motion field can be approximated as a linear combination of the individual forces.

**Figure 7:**
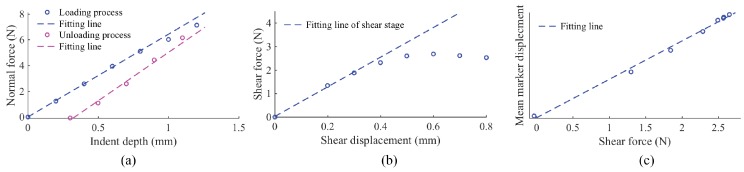
(**a**) when an indenter pressed on the GelSight surface in normal direction, the force is in linear relationship with the indenting depth, but the unloading curve is different from the loading curve due to the viscoelasticity; (**b**) when an indenter moves on GelSight in the shear direction, the shear force is initially proportional to the shear displacement of the indenter, but then grows slowly and comes to a constant value when partial slip or slip occur; (**c**) under the shear force, the average marker displacement is proportional to overall shear force, regardless of whether it is in the shear stage or slip stage.

The force-deformation curves of the fingertip GelSight (when elastomer not glued on sensor) are shown in Figure 7. In Figure 7a, the gap between the loading and unloading curves is caused by the viscoelasticity. Figure 7b shows that when the shear load is small, the force is linear to the load displacement; when the load increases, partial slip or slip occurs, which stops the shear force from growing. Figure 7c is from the same shear experiment, and it shows that the average motion magnitude of the markers within the contact area is proportional to the shear force, regardless of whether slip or partial slip occurs. In fact, the linear relationship remains even before force reaches equilibrium.

On the other hand, the linear relationship between force and marker motion only exists when the contact geometry remains unchanged. When contacting an object of another shape, there is still a linear relationship between the force and marker motion, but the parameters change. To achieve a shape independent relationahip between force and displacement, we turn to machine learning. A method to measure the force when contacting unknown geometries is to apply Convolutional Neural Networks (CNN) on the GelSight image. CNNs have proved very effective in extracting features from the nonlinear and high-dimensional data, like images. The GelSight output is similar to normal computer images in that it is also high-dimensional, noisy, and highly nonlinear. It can be expected that the CNN can also pick up the useful features regarding to contact force from GelSight data.

### 3.7. Marker Motion for Slip Detection

The GelSight sensor can detect signals related to slip and incipient slip by analyzing the distribution of the markers’ motion. When the markers in the peripheral contact area moves significantly less than the markers in the central or along the shear directions, it is very likely that slip has occurred or is going to occur soon. The rule holds for the GelSight sensor with both flat and curved surface. The physical foundation of this measurement is that, when slip going to occur under a soft contact condition, it starts from the peripheral contact area, which results in the relative displacement between the sensor surface and the object in the area, but the central area is still ‘stuck’ to the contact surface until the overall slip occurs. Ito et al. [22] used the similar feature to detect slip with a vision-based soft sensor.

**Figure 8:**
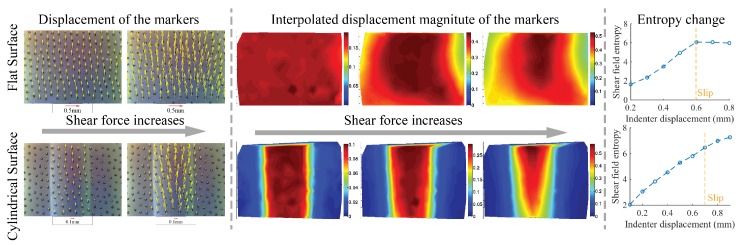
The change of the marker displacement field with the increase of shear force. The degree of partial slip also increases as the force grows, and it starts from the peripheral contact area, which results an inhomogeneity in the marker displacement field. We can measure the inhomogeneity with the entropy of the markers’ displacement, which keeps increasing. The experiment is conducted with a fingertip GelSight sensor [30] whose surface is flat, (Figures adapted from [27] (Got copyright permission from IEEE)).

Examples of how the displacement field changes as the shear load increases are shown in Figure 8 (experiments done with the fingertip GelSight sensor with flat sensor elastomer piece). The degree of partial slip can be inferred from the inhomogeneity degree of the marker displacement distribution. We use the entropy to describe the inhomogeneity degree of the displacement field. The entropy of a histogram X is

(5) $$H\left(X\right)=-\int_{X}p\left(x\right)\log p\left(x\right)dx.$$

The entropy *H* increases as the partial slip degree increases, shown in the last column in Figure 8. To prevent the slip occurrence, a possible way is to set a warning threshold on *H*.

**Figure 9:**
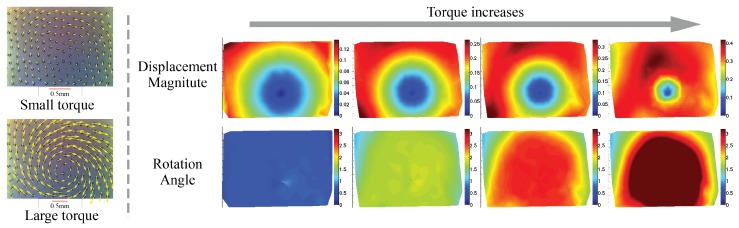
The change of the marker displacement field and rotational angle with the increase of in-plane torque. The contact surface is flat. When the torque grows large, partial rotational slip or full slip occurs, starting from the peripheral area. The partial rotational slip results in the inhomogeneous distribution of the markers’ rotational angle. The experiment is conducted with a fingertip GelSight sensor [30] (Got copyright permission from IEEE) whose surface is flat, and the object in contact is a flat surface.

Apart from translational slip, rotational slip is also commonly seen in grasping. Similarly, partial rotational slip occurs before the total slip occurs, and it starts from the peripheral area. The partial slip can be inferred from the inhomogeneous distribution of the markers’ rotational angles along the rotational center, where the markers in the peripheral area make much less angles. Figure 9 shows examples of how the marker motion and rotation angle change as the in-plane torque increases.

The marker motion analysis for detecting slip or partial slip is most effective for contacting the objects with flat surfaces or small curvatures, when the contact surface is large enough to contain multiple markers, and the object motion can be barely detected by the motion of contact geometry. For objects with complicated shapes or obvious textures, slip can be more easily detected by comparing the motion of the marker and the motion of the shapes. This is equivalent to comparing the shear or rotation motion of the sensor surface and the motion of the object. In reality, the success rate for slip detection on general objects could be largely increased when combining the multiple cues. Detailed experiments are shown in [29].

## 4. Design and Fabrication of GelSight

### 4.1. The Desktop GelSight Sensor

The first GelSight sensor Johnson and Adelson [7] is shown in Figure 2: the sensor is in a shape of a cubic box with the side length of 30 cm, and is made of standard building frames and dark acrylic boards. The sensing elastomer is placed on the top, and the reflective coating of the elastomer is semi-specular, made from bronze flake pigment. The reflective coating has a sharp response to surface textures. A machine vision camera is placed in the bottom. Three LED arrays, covered by the filters of red, green, and blue, are placed under the sensor surface, making the lights illuminate at the elastomer with an angle of 60°. The LEDs have lenses on the top, so that the lights getting out of the LEDs are in small angles. The sensing range of the sensor is about 60 mm × 40 mm, which is only a small part in the central of the sensor’s top surface.

**Figure 10:**
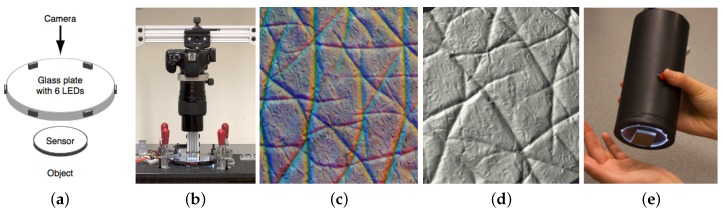
The GelSight device with matte lcoating and side illumination [8] (Got copyright permission from Association for Computing Machinery). (**a**) the schematic of the optical system: six LEDs with a single color (white) is evenly distributed on the side of the supporting glass, and the sensor elastomer is in the central area of the supporting plate; (**b**) the bench setting of the design: a digital single-lens reflex (SLR) camera and macro lens is used to capture the high-resolution images on the sensor surface; (**c**) the captured image of human skin. (**d**) the reconstructed geometry of the skin from (**c**); (**e**) the portable design of the sensor.

Johnson et al. [8] propose an improved version of the sensor with better spatial resolution. For the sensor coating, they choose a fine and diffuse surface made of silver powder with particle size of one micro and in spherical shape. The diffuse reflectance makes the coating have a more uniform response to a broader range of surface normals, thus the sensor could capture a more accurate geometry. The diffuse coating makes more distinctive reflection under a side lighting conditions, which means that the incoming lights are expected to be nearly parallel to the sensor surface. To do this, the researchers mount the LEDs on the side of a clear glass plate, and mount the sensing elastomer in the central part of the plate, as shown in Figure 10a. In this case, the lights from LEDs are guided by the glass plate and is in an approximately parallel angle when entering the elastomer. The researchers arrange six LEDs around the glass plate, all in white color. The six LEDs light up asynchronously, and the camera captures six images under each LED lighting. The design increases the precision of the measurement of surface normal. With the same optical design, the authors made a bench configuration sensor (Figure 10b) and a portable configuration (Figure 10e), and both sensors can get a high resolution geometry of the contact surface. However, the asynchronous setting of the lights takes longer time to obtain one geometry measurement, so that the sensor can not be used to measure real-time dynamic tactile information.

### 4.2. Fingertip GelSight Sensors

A qualified tactile sensor for robot hand should be small in volume and should be able to acquire online signal and processing. For the GelSight sensor, the major design challenge is to reduce the size of the illumination system while maintaining the illumination quality, which means the lights should enter the sensing elastomer in the concentrative and desired angles. However, on the other hand, for most of the robotic tasks, the required precision of the geometry measurement is not very high.

In this section, we introduce two versions of the fingertip GelSight sensors that are designed for robot grippers. The illumination of the sensors are of different colors, and the embedded camera is a commercialized USB web-camera (C310 from Logitech, Lausanne, The Switzerland). The camera can be easily connected to a computer through USB, and capture images at 30 Hz with a resolution as high as 1920 × 1080 (we mostly use the resolution 640 × 480 because the driver is more widely supported). Therefore the sensors could measure the online tactile images and reconstruct the 3D geometry. (Note, however, that this sensor, like other USB two sensors, uses H.264 encoding, which introduces significant image latency). The sensing field is a 18 mm × 14 mm area in the center of the sensor, and the spatial resolution is around 20 to 30 microns. We also make the sensing elastomer on the sensor in the domed shape, which enables the contact over a wider set of conditions.

**Figure 11:**
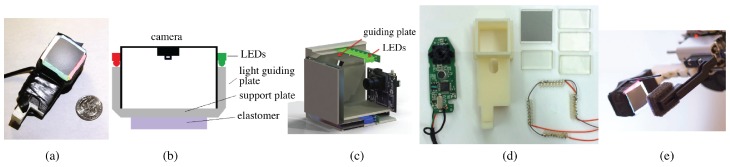
Fingertip GelSight sensor introduced in [28,30] (Got copyright permission from MIT and IEEE): the illumination has four colors (red, green, blue and white), and the entire sensing elastomer through the guiding plates are made of clear acrylic boards. The sensing elastomer coating in this design is the semi-specular coating. (**a**) picture of the sensor; (**b**,**c**) schematic of the sensor; (**d**) the parts of the sensor before assembling; (**e**) integration of the sensor into the Baxter Robot’s gripper.

The first version is built by Li et al. [30], and is designed for a Baxter Robot gripper (Rethink Robotics, Boston, MA, USA) (Figure 11a,e). The sensor is close to a cubic shape, with the compact size of 35 mm × 35 mm × 60 mm. The design uses perpendicular acrylic plate sets to guide the lights from the top to the sensing elastomer in the bottom, as shown in Figure 11b. The sensing elastomer has semi-specular coating to reveal the details and small fluctuations on the object’s surface. After the internal reflection in the acrylic plate, the lights will be at the angles close to the parallel direction of the elastomer surface. The LEDs on the four sides of the sensors are in the form of a line array (1 × 8), and are of four colors: red (R), green (G), blue (B) and white (RGB). The hue and saturation of each pixel indicates the direction (yaw angle) of the surface normal, since the light sources from different directions are different in color, and the intensity corresponds to the magnitude (pitch of surface normal). The parts of the sensor are displayed in Figure 11d: the support structure is 3D printed with non-transparent materials, which holds the camera and the acrylic guiding plates, as well as providing the mounting structure to the robot gripper. The clear acrylic plates are cut by a laser cutter, and one side is manually ground to 45° to change the light direction. The LEDs are manually soldered into compact arrays, and glued to the top side of the support, just in front of the acrylic guiding plates.

This sensor has two major deficiencies: firstly, although the sensor is very sensitive to small curvatures, the measurement of surface normal is not precise because of the non-parallel illumination and semi-specular coating of the gel. The size reduction decreases the illumination quality. Secondly, the fabrication of this sensor is over-complicated. Too much accurate operation is required by manual work, so that the product is hard to be standardized, and the fabrication is time-consuming.

**Figure 12:**
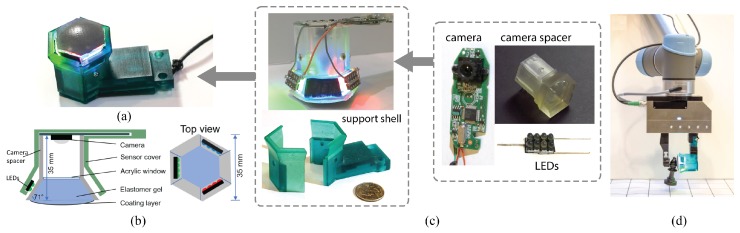
The new GelSight fingertip sensor introduced in [29] (©2017 IEEE). The sensor has three LED arrays of different color to illuminate the elastomer surface from a tilted angle of 71°, and a USB web cam to capture real-time image from the sensor. (**a**) the picture of the sensor; (**b**) the schematic diagram; (**c**) the parts of the sensor; (**d**) the sensor mounted on a WSG-50 parallel gripper (Weiss Robotics, Ludwigsburg, Germany).

In 2017, Dong et al. [29] proposed another version of the fingertip GelSight sensor (Figure 12a), with largely improved precision of shape measurement and simplified fabrication process. The new sensor is of approximately the same size and spatial resolution with the previous fingertip sensor, but is in a hexagonal shape, and has a new illumination system using LEDs with three colors (RGB). The new LEDs (Osram Opto Semiconductor Standard LEDs-SMD, Sunnyvale, CA, USA) have small collimating lenses, and the emitted light are within a viewing angle of 30°. They are tightly arranged into 2×4 arrays with a customized circuit boards, and are tilted at the angle of 71° to the sensing elastomer surface from the sides. The LED and elastomer are supported by a semitransparent tray, which homogenizes the LED light while allowing a high transmission rate. The sensor coating is a matte surface, and the matte coating as well as the entire illumination system favor a more accurate surface normal measurement.

Most of this sensor’s parts are 3D printed with a Formlab 2 printer (Formlabs Inc., Somerville, MA, USA), and the clear acrylics are cut by a laser cutter. The parts are shown in Figure 12c. The base part, a semitransparent hexagonal plastic tray and camera spacer, is printed with clear resin. The tray is filled with a piece of clear acrylic sheet, and the sensing elastomer is glued on the acrylic sheet. The camera is mounted on the top of the hexagonal tray to capture the deformation of the elastomer. A support shell, 3D printed with ‘tough’ resin, covers the circuits and lights, as well as providing the mounting structure to mount the sensor to a WSG-50 robot gripper. The drawings for the 3D printing parts are open-sourced at [33]. The fabrication of the new sensor is highly standardized, and manual labor is significantly reduced.

### 4.3. Fabrication of the Sensor Elastomer

The sensing elastomer used on GelSight is made of two parts: the clear elastomer sheet as the base, and the reflective membrane. The elastomer sheet can be made from many kinds of silicone or thermoplastic elastomer (TPE), as long as it is transparent and has good deformability. However, the reflective membrane is essential for the sensor’s signal quality. For making good signal quality, the membrane must be fine, uniform, thin, smooth, firm, and light-blocking. The fineness and thickness of the membrane will influence the resolution of the shape measurement, and if the membrane is not uniform or smooth, the tactile images will contain noise from the bumps on the surface. Finally, we must glue the elastomer on the sensor’s supporting plate to reduce the residue force during the contact. The glue we commonly use is a double-side adhesive silicone sheet.

The typical fabrication of the sensing elastomer contains three steps: making the transparent elastomer base, printing the markers, and painting the reflective coating over the markers and base. To make the elastomer, we choose some commercialized polymer that comes in the fluid phase. We pour the liquid material in the mold, either a flat tray or a concave mold, depending on what kind of sensor we need. Then, we demold the elastomer after it solidifies. The elastomer we use the most is the XP-565 from Silicones, Inc. (High Point, NC, USA). It comes in two liquid parts, and after mixing the two parts at some specific portion, we pour the silicone in the molds, and it solidifies within several hours under room temperature, or within 15 min in an oven at the temperature of 100 °C. The hardness of the elastomer can be adjusted by changing the mixture portion of part A and B.

We paint the markers on the elastomer using water transfer paper from Lazertran. We firstly print the designed marker patterns on the transfer paper using a laser printer, and then lay the printed side on the elastomer, and wet the paper from the back. The printed markers will transfer to the elastomer after we peel off the transfer paper.

There are three kinds of coating pigments that we use: the bronze flake and aluminum flake for semi-specular coating, and 1 μm spherical aluminum powder for matte coating. Examples of the different coatings are shown in Figure 4. The different coatings are good for different applications, and they require different illumination systems. There are two kinds of methods to make the coating: one is using the airbrush to spray the dissolved pigment on the elastomer base, and the other one is to directly brush the pigment powder on the elastomer base.

The semi-specular paint coating is made by airbrush. Then, we disperse the metal flake pigment in some silicone paint base (Cirius from ArtMolds) and organic solvent (such as toluene, d-limonine, methyl ethyl ketone (MEK)), and use an airbrush to evenly spray the solution on the elastomer surface, layer by layer. The airbrush can make the coating thin and even, but it takes a very long time for a fine piece of work, and we must pay close attention to removing the dust stuck in the coating. Brushing the pigment powder directly is much easier, but it only applies for pigment that comes as dry powders. Those powders will naturally stick on the silicone base. We use a make-up brush to evenly spread the powders on the surface, and then coat another thin protective silicone layer on the top. The simplest way to coat the layer is to pour the diluted fluid silicone on the painted elastomer.

## 5. Evaluation

In this section, we take the new compact GelSight sensor mentioned in [29] as an example to evaluate the sensor’s performance in estimating object shapes and contact force.

### 5.1. Evaluation of Shape Measurement

**Figure 13:**
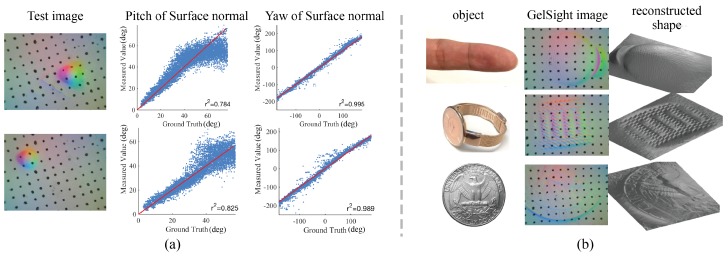
Evaluation on the shape measurement of fingertip GelSight (Figures adapted from [29] (©2017 IEEE)). (**a**) the measurement of surface normal, when there the sensor is contacting a small ball. The plots compare the measured surface normal value and the ground truth, where the red line marks equal relation. The pitch can be considered as the magnitude of geometry gradient of the contact surface, and the yaw can be considered as the planar direction of the gradient; (**b**) examples of reconstructed 3D geometry when contacting different objects.

For evaluating GelSight’s geometry measurement, we firstly evaluate the sensor’s precision in measuring the surface normal. We use the same images for calibration: the GelSight images of a small ball (d=3.96 mm) pressed on the sensor. The ground truth of the surface normals can be calculated from the known geometry of the ball, and we measure the surface normal using the calibrated lookup table, as introduced in Section 3.3 and Section 3.5. Examples of the results are shown in Figure 13a. In the figures, we compared the measured values and the ground truth, of the pitch and yaw angles of the surface normal. In the x−y plane of the sensor, the pitch angle can be considered as the geometry’s spatial gradient, and the yaw angle can be considered as the gradient direction. The figures show that the measured value are correlated with the ground truth, especially when the gradient is low or medium; the measurement of yaw angle, or the gradient direction, is much more precisely measured. The figures also indicate that there is some spatial variance of the sensor’s measurement, but can be accepted for an approximate measurement. Figure 13b gives some examples of the reconstructed geometry of common objects. The ground truth of the geometry is hard to get, but the reconstructed 3D structures capture both the overall shape and local textures of the objects.

### 5.2. Evaluation of Force Measurement

**Figure 14:**
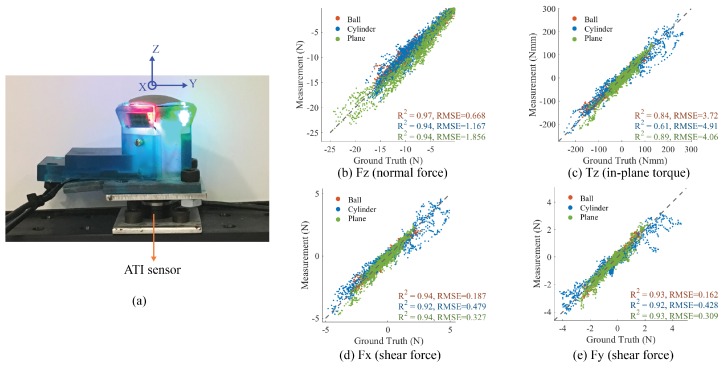
Evaluation on the force measurement of fingertip GelSight with simple but unseen objects. (**a**) experiment setup, where the GelSight is fixed on a ATI Nano-17 force-torque sensor, which is used to measure ground truth. In the experiments, we manually contact the GelSight surface with different objects and different loads. (**b**–**e**) experiment results of the force torque measurement with different unseen objects, including a ball, a cylinder and a flat plane. The gray dashed line denotes the 1:1 plot where the measurement meets the ground truth.

The GelSight sensors measure force and torque using the displacement field of the markers on the surface. The motion of the markers correspond to the lateral displacement of the sensor’s surface, and the deformation of the elastomer can infer contact force. However, the relationship between the markers’ motion and the contact force or shape is nonlinear when the contact geometry is unknown. For general situations, we use deep neural network to measure the contact force and in-plane torque from the GelSight images. In this section, we evaluate the fingertip GelSight sensor’s ability in measuring force and torque with Convolutional Neural Network (CNN) in basic cases. In this preliminary experiment, we train the force measurement neural network with the data of GelSight contacting objects with some basic shapes, including spheres, cylinders and flat plane, and then test the network’s performance on measuring the force when contacting other similar but unseen objects The forces and torque we try to measure is the normal force, shear force and direction, and in-plane torque (the torque along the *z*-axis in Figure 14a).

The experimental setup is shown in Figure 14a: a fingertip GelSight sensor is mounted on an ATI Nano-17 force/torque sensor on a fixed table, and we manually push or twist different objects against the GelSight sensor with different force amounts and directions, so that the force and in-plane torque on the GelSight surface is equal to the load on the ATI sensor, and we use the measurement from the ATI sensor as the ground truth. The objects include six balls (diameters from 12 mm to 87 mm), five cylinders (diameters from 10 mm to 70 mm), and two flat surfaces of different rigid materials. The total size of the training dataset is around 28,815. We only use the data in the loading process to reduce the influence of viscoelasticity.

The CNN model for measuring force and torque is adjusted from VGG-16 net [34], pre-trained on the computer vision dataset ImageNet [35]. We replace the network’s last fully-connected layer with an output layer of four neurons, corresponding to the forces and torques in four axes (Fx, Fy, Fz, Tz). The input of the network is the three-channel difference image of the current GelSight image and the initial image, when nothing is in contact. We train the network with the mean squared error loss function for the regression problem. To test the model, we use the GelSight data of contacting three novel objects: a ball (d= 25 mm), a cylinder (d= 22 mm), and a flat plane. The test set contains 6705 GelSight images under different forces and torques.

The comparison between the output of the neural network and ground truth from the force/torque sensor is summarized in Figure 14b–e. The coefficient of determination ($R^{2}$) and root mean square error (RMSE) for the results of three different objects are also listed in the figure. The plots show that the model output of forces and torques by GelSight sensor are correlated to the ground truth measured by the force torque sensor. For the force measurements, $R^{2}$ is higher than 0.9. The results also show that the GelSight measurement of force can be robust regardless of the geometry of the contact objects.

However, for measuring the contact force and torque, the CNN methods have some deficiencies—that it can not well generalize the force-related information from the GelSight images—which costs most of the measuring error. On the one hand, the prediction from CNN highly relies on the training data, and to make robust measurement, the training data should contain the contact cases with objects of all shape categories and all contact conditions. On the other hand, for the given training set and test set, the measurement is still influenced by the contact geometry. We found that if we align the measurement in the chronological order, it well matches the ground truth qualitatively at all times, but the measurement at some entire contact sequences is worse than the others. In general, the GelSight images within the same contact sequence is of similar contact conditions, and the geometry is similar, while the CNN may make some biased measurement towards the certain contact geometry. In the long run, for making a good force measurement with GelSight, we should either collect a more comprehensive dataset (simulation methods could be applied), or choose some other methods that can better extract force information from GelSight images.

## 6. Application

With the high-spatial resolution of shape measurement, GelSight is proved to be effective in many robotic tasks that are very challenging for traditional tactile sensors.

**Figure 15:**
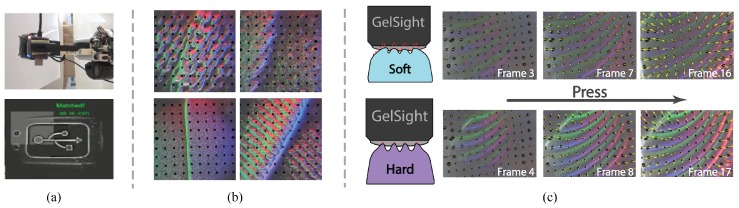
Examples of GelSight applications. (**a**) Li et al. [30] (Got copyright permission from IEEE): a robot inserts a USB cable into the socket, while GelSight image helps the robot to locate the in-hand position of the USG plug; (**b**) GelSight can differentiate difference fabrics from their textures (images are enhanced for display purpose). The shape of the foldings can also reveal other material information, such as the thickness, stiffness [36] (Got copyright permission from IEEE); (**c**) When pressing on a deformable object [37] (Got copyright permission from IEEE), the GelSight sequence can infer hardness information from the change of the object shapes and contact force.

Li and Adelson [38] show that GelSight can recognize materials from textures with very high precision. Figure 15a gives some examples of the GelSight images when pressing on different fabrics. Although the fabrics are in the same object category with similar physical properties, GelSight can discriminate them by recognizing the high-resolution texture of the textile type. Additionally, because GelSight can measure the shapes of the objects, the geometry of the folds on the fabrics can be used to infer other properties such as thickness or stiffness.

At the same time, GelSight can sense other object properties as well. Yuan et al. [37,39] show that GelSight can measure the hardness of objects from a dynamic image sequence when the sensor is pressed against the object. The sensor records how the shape and force of the sensor surface changes as the contact proceeds. Objects with different hardness may make different deforming patterns, since softer objects have more deformation in geometry in the contact process. Figure 15b shows some examples. By using neural networks, the GelSight sensor can estimate hardness of the objects with unknown shapes, under a loosely controlled contact condition, while the contact force and trajectory are unknown.

GelSight can assist with multiple manipulation tasks as well. As indicated in Section 3.7, the GelSight sensor can estimate the slip and incipient slip state from the stretching of the surface. Additionally, it can detect slip by measuring the relative movement of the objects on the sensor. Dong et al. [29] presents experiments of robot gripper successfully detecting or predicting slip from multiple cues when grasping different kinds of objects. Li et al. [30] shows the usage of GelSight in the USB insertion task: the GelSight sensor measures the USB pattern on the connector, thus calculating the in-hand position of the connector. The robot then refines the manipulation position using the in-hand position, which increases the success rate of the inserting task. Izatt et al. [40] further shows that GelSight can serve as a supplemental sensing modal to picture a much more precise point-cloud of the area within the grippers.

## 7. Conclusions

Tactile sensing is an important modality for robots, and one of the major challenges for robotic tactile sensing is to develop sensor hardware that can obtain adequate tactile information for multiple perception and manipulation tasks. We provide a possible solution by developing a novel tactile sensor, GelSight, which can measure high-resolution geometry, as well as information about force and shear. In this paper, we review the principles, algorithms, design, fabrication process and performance evaluation of some different versions of the GelSight sensor. Apart from measuring geometry and force, GelSight can also indicate other information, such as slip or incipient slip. The fingertip version of GelSight has been successfully applied on robotic grippers, and the new design makes the sensor fabrication and the data accessibility much more convenient. With the tactile information provided by GelSight sensor, a robot will be able to perform much better in multiple tasks related to both perception and manipulation.

## Figures and Tables

**Table 1:** The minimum distinguishable force of the fingertip GelSight (Setup shown in Figure 14a, using the shape measurement and marker measurement respectively. The sensor elastomer has a neo-Hookean coefficient of 0.145 MPa. Since we use an ATI Nano-17 force/torque sensor to calibrate GelSight force measurement, and minimum distinguishable normal force for the setup is 0.05 N, we are not able to get finer measurement of the minimum force when using marker measurement.

Contact Surface Type	Rigid		Soft (Shore 00-10)	
Contact area	30 mm${}^{2}$	Flat (>2 cm${}^{2}$)	30 mm${}^{2}$	Flat (>2 cm${}^{2}$)
Using shape measurement	<0.05 N	<0.05 N	<0.05 N	0.08 N
Using marker measurement	<0.05 N	<0.05 N	<0.05 N	<0.05 N
